# Correlation of angiotensin I-converting enzyme gene insertion/deletion polymorphism with rheumatic heart disease: a meta-analysis

**DOI:** 10.1042/BSR20160151

**Published:** 2016-11-22

**Authors:** Yulong Tian, Zhongchun Ge, Yuliang Xing, Yan Sun, Jie Ying

**Affiliations:** * Department of Cardiology, People's Hospital of Xuyi, Xuyi 211700, Jiangsu, P.R. China; † Department of Infectious Diseases, People's Hospital of Xuyi, Xuyi 211700, Jiangsu, P.R. China

**Keywords:** angiotensin I-converting enzyme, meta-analysis, polymorphism, rheumatic heart disease

## Abstract

Rheumatic heart disease (RHD) is a serious cardiovascular disorder worldwide. Several articles have reported the effect of angiotensin I-converting enzyme gene insertion/deletion (ACE I/D) polymorphism in RHD risk. However, the results still remain inconsistent. The objective of the present study was to assess more precise estimations of the relationship between ACE I/D variant and RHD susceptibility. Relevant case–control studies published between January 2000 and 2016 were searched in the electronic databases. The odds ratio (OR) with its 95% confidence interval (CI) was employed to calculate the strength of the effect. A total of nine articles were retrieved, including 1333 RHD patients and 1212 healthy controls. Overall, our result did not detect a significant association between ACE I/D polymorphism and RHD risk under each genetic model (*P* > 0.05). Subgroup analysis by ethnicity showed no positive relationship in Asians as well (*P* > 0.05). With respect to the severity of RHD, our result found that the frequency differences between mitral valve lesion (MVL), combined valve lesion (CVL) and healthy controls were not significantly different. Furthermore, no significant association was found between female, male RHD patients and the controls regarding to the ACE I/D polymorphism. In conclusion, our result indicated that ACE I/D polymorphism might not be a risk factor for RHD progression based on the existing research results. Additional well-designed studies with larger samples are still needed to confirm these findings.

## INTRODUCTION

Rheumatic heart disease (RHD) is a disease that results from an abnormal autoimmune response to a group A streptococcal infection in a genetically susceptible host [1]. It still is a major health burden in most developing countries as well as sporadically in developed economies, leading to significant morbidity and mortality [2]. Each year, at least 282000 people develop RHD worldwide [3–5]. According to the Global Burden of Disease study (GBD), there were approximate 34.2 million people with RHD resulting in 345110 deaths [6], and 10.1 million disability adjusted life years lost in 2010 [7]. The pathogenesis of RHD is complex and not fully understood [8]. The treatment for RHD remains more difficult [9]. Although echocardiographic diagnosis for RHD has greatly improved the sensitivity over the past 5 years, concern about the specificity of echocardiography and the interpretation of minor abnormalities has posed new challenges [10,11]. Furthermore, it has been estimated that as many as 50% or more of patients are unaware of their RHD and as many as 70% do not receive secondary prophylaxis [12]. Therefore, it is urgent to identify some biomarkers to predict this disease and guide the therapeutic strategies.

Epidemiologic studies have shown that RHD depends on several host factors that mediate the inflammatory and heart-tissue driven autoimmune response, and molecular mimicry and genetic predisposition are involved in autoimmune reactions [13,14]. Angiotensin-converting enzyme (ACE), a zinc-dependent peptidase, is an important component of the renin–angiotensin system (RAS) responsible for converting angiotensin (Ang) I to vasoconstrictor Ang II [15]. It is located on human chromosome 17q23 and has been implicated in many physiologic processes such as blood pressure control, haematopoiesis, reproduction, renal development, renal function and the immune response [16]. Inhibition of ACE activity could suppress tumour growth and angiogenesis, and reduce cardiovascular deaths [17,18]. The presence of a common variant, the insertion/deletion (ACE I/D, rs1799752) polymorphism in intron 16 of the ACE gene, could influence the serum and tissue ACE activity, accounting for half the variance of serum enzyme levels. The I allele, which represents an insertion of 287-base pair (bp), is associated with lower serum and tissue, and the deleted form of the variant (D allele) is associated with higher circulating and tissue ACE activity [19,20]. This genetic polymorphism was associated with coronary artery disease [21], systemic lupus erythematosus [22] and cancer risk [23].

Although several studies have identified the effect of angiotensin I-converting enzyme gene insertion/deletion (ACE I/D) polymorphism in RHD risk, the results still remain inconclusive. In addition, the incidence of RHD varies among different populations [24], and the distribution of ACE I/D polymorphism has been reported to differ between various ethnic populations [25]. Therefore, we conducted this meta-analysis to systematically review all the published articles and to reassess whether ACE I/D variant was associated with RHD risk or not.

## MATERIALS AND METHODS

### Search strategy

We conducted a comprehensive literature search to retrieve eligible studies published between January 2000 and 2016 in the following electronic database of PubMed, Web of Science, Medline, Embase, CNKI (China National Knowledge Internet) and Wanfang. The MeSH terms were: ‘RHD’, ‘angiotensin I-converting enzyme or ACE’, ‘insertion/deletion or I/D’, ‘polymorphism or mutation or variant’ as well as their combinations. We manually searched the references of related articles to obtain additional sources. Articles were only restricted in English and Chinese languages. When the same authors or laboratories reported this issue on the same populations, only the recent full-text article was included.

### Inclusion and exclusion criteria

The inclusion criteria were as follows: (1) case–control studies that focused on the association between ACE I/D polymorphism and RHD risk; (2) patients with RHD were confirmed by echocardiography criteria [26]; the controls were age-, gender- and race-matched participants with normal echocardiograms and had no family history of cardiac illness or autoimmune disease; (3) the frequencies of alleles and genotypes in each article were available to extract and (4) the results were expressed as odds ratio (OR) with its 95% confidence interval (CI). The exclusion criteria were (1) without control group; (2) data not available; (3) with duplicate data and (4) review reports or conference papers.

### Data extraction

Two authors estimated the quality of the included studies independently. The methodological quality for each single study was estimated by a modified strengthening the reporting of observational studies in epidemiology (STROBE) quality score system [27]. Thirty-nine assessment items matching the quality appraisals were used, with scores ranging from 0 to 39. Score of a single study was more than 19 that considered as moderate or high-quality article and was finally included in this meta-analysis. Any disagreement was subsequently resolved by discussion with a third author to obtain a final consensus. The following information was extracted from each included article: the name of first author, published year, country, ethnicity, mean age, sample size, genotyping method, score of each include study, frequencies of genotypes and alleles, risk genotypes and evidence of Hardy–Weinberg equilibrium (HWE) in controls.

### Statistical analysis

Statistical analyses were conducted in Review Manager (version 5.3, The Cochrane Collaboration). The strength of the association between ACE I/D polymorphism and RHD susceptibility was measured by ORs with 95% CI. The significance of the pooled ORs was determined by the *Z* test, with a *P*-value less than 0.05 considered statistical significance. The HWE in controls were examined by HWE test [28]. The allelic model (D versus I), homologous model (DD versus II), heterogeneous model (ID versus II), dominant model (DD + ID versus II) and recessive effect (DD versus ID + II) were examined to evaluate the effect of I/D variation of RHD risk. The between-study heterogeneity was determined by the *I*^2^ test and the *Q*-statistic test. The fixed-effect model was used when the *I*^2^ for the *I*^2^ test was less than 50% and the *P*-value for the *Q*-test was more than 0.10; otherwise, the random-effect model was used. The evidence of publication bias was assessed by visual funnel plot inspection.

## RESULTS

### Baseline characteristics of included studies

**Figure 1:**
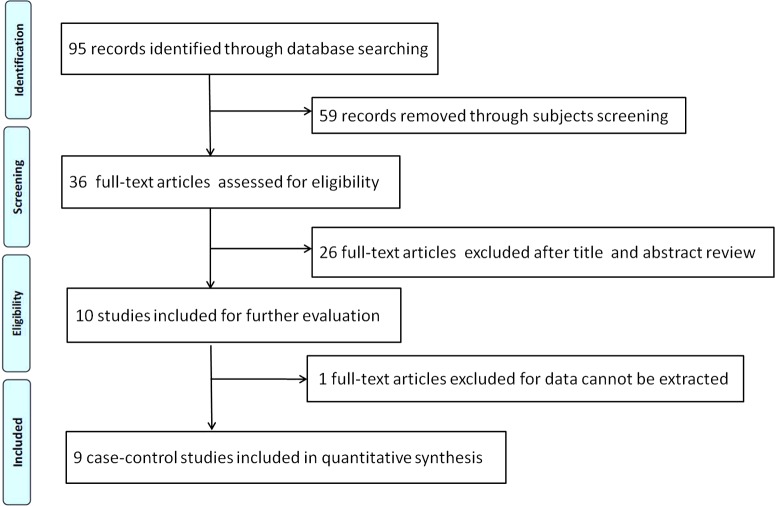
Flow chart of selection process in this meta-analysis

We firstly identified 95 articles, after applying the inclusion and exclusion criteria, nine relevant articles (eight were written in English and one in Chinese) were finally screened out. Figure 1 shows the flow diagram of selection process. Overall, a total of 2545 subjects were involved in this meta-analysis, including 1333 RHD patients and 1212 controls. The nine studies (eight were performed in Asian population and one in African population) were conducted in seven countries: Turkey [29,30], Egypt [31], India [32], China [33,34], Kazakhstan [35], Kingdom of Saudi Arabia [36] and Pakistan [37]. The ACE I/D variant was measured by PCR. The distribution information of genotypes in controls were all in accord with HWE (*P* > 0.05) except the study conducted by Rehman et al. [37]. Table 1 presents the main characteristics of included studies in this meta-analysis. Table 2 lists the distribution of alleles and genotypes of ACE I/D polymorphism in each study.

**Table 1:** Main characteristics of included studies in this meta-analysis (NS, not significant)

				Mean age		Sample size				
								
First								Genotyping	Risk	Scores of
author	Year	Country	Ethnicity	Cases	Controls	Cases	Controls	method	genotypes	studies
Atalar E	2003	Turkey	Asian	21±6	25±6	126	39	PCR	DD	31
Chou HT	2004	China (Taiwan)	Asian	51.0±12.2	49.8±16.5	115	100	PCR	I, II	25
Davutoglu V	2005	Turkey	Asian	40.3±14.7	43.4±13.4	82	154	PCR	II	34
Morsy MMF	2011	Egypt	African	9.5±2.2	9.1±2.6	139	79	PCR	DD	35
Gupta U	2013	India	Asian	35.41±12.69	37.36±13.41	300	200	PCR	D, ID, DD	29
Zhang T	2013	China	Asian	48±10	49±10	246	223	PCR	I, II	20
Bakhtiyarova GK	2014	Kazakhstan	Asian	42.83±1.06	40.24±0.87	70	68	PCR	NS	28
Al-Harbi KM	2015	Kingdom of Saudi Arabia	Asian	19.4±5.2	20.6±4.5	99	145	PCR	DD + ID	33
Rehman S	2015	Pakistan	Asian	31±14.10	18.3±12.7	156	204	PCR	I, II	37

**Table 2:** The alleles and genotypes distribution of ACE I/D polymorphism in each included study in this meta-analysis

	Cases					Controls					
First author	II	ID	DD	I	D	II	ID	DD	I	D	HWE
Atalar E	22	52	52	96	156	10	21	8	41	37	0.883
Chou HT	55	41	19	151	79	30	52	18	112	88	0.859
Davutoglu V	26	25	31	77	87	28	69	57	125	183	0.679
Morsy MMF	43	59	37	145	133	29	39	11	97	61	0.935
Gupta U	101	167	32	369	231	92	94	14	279	121	0.307
Zhang T	124	97	25	345	147	88	109	26	285	161	0.674
Bakhtiyarova GK	18	43	9	79	61	24	34	10	82	54	0.936
Al-Harbi KM	4	45	50	53	145	19	62	64	100	190	0.811
Rehman S	27	100	29	154	166	13	140	51	166	242	0.000

### Association between ACE I/D variant and RHD susceptibility

**Figure 2:**
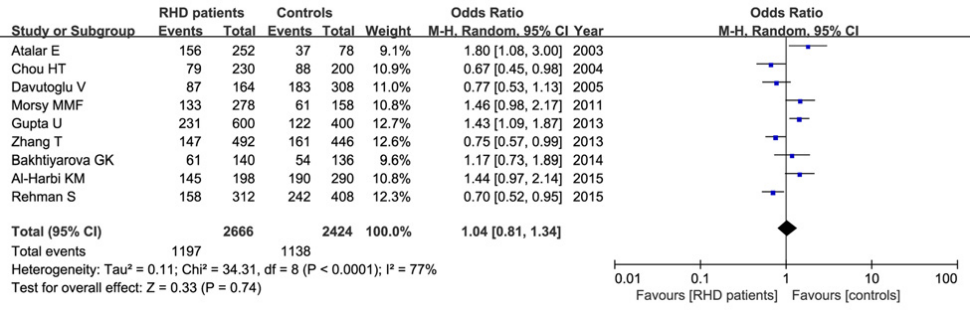
Meta-analysis of the relationship between the ACE I/D polymorphism and RHD risk under the allelic model (D versus I)

Table 3 provides the meta-analysis findings of the associations between ACE I/D variant and RHD risk. The between-study heterogeneity was calculated, and the heterogeneity was existed in most genetic comparisons. In this meta-analysis, we detected that the distribution of D allele in cases in each article ranged from 29.9% to 73.2%. Our result found that the frequency of D allele was a little lower in RHD patients than that in healthy controls (44.9% versus 46.9%), and the statistical analysis showed no significant difference in the rate of allele mutation between RHD cases and controls (D versus I: OR=1.04, 95% CI=0.81–1.34, *P*= 0.74) in the random-effect model as shown in Figure 2. This insignificant relationship was observed in other genetic models as well in the random- or fixed-effect model (*P* > 0.05, Table 3). Subgroup analysis by ethnicity showed that there was no positive association between ACE I/D variant and RHD susceptibility in Asians under the five genetic models (*P* > 0.05).

**Table 3:** Meta-analysis results of ACE I/D polymorphism on RHD risk in total and subgroup analysis *N*, number of included studies; R, random-effect model; F, fixed-effect model.

			Test of association		Test of heterogeneity		
Group	Comparisons	*N*	OR (95% CI)	*P*	Ph	*I*^2^ (%)	Model
Total	D versus I	9	1.04 (0.81, 1.34)	0.74	<0.0001	77	R
	DD versus II		1.11 (0.63, 1.93)	0.72	<0.0001	76	R
	ID versus II		0.86 (0.54, 1.35)	0.51	<0.0001	79	R
	DD + ID versus II		0.94 (0.60, 1.48)	0.79	<0.00001	82	R
	DD versus ID + II		1.15 (0.93, 1.41)	0.20	0.08	43	F
MVL	D versus I	6	1.02 (0.74, 1.41)	0.89	0.0008	68	R
	DD versus II		1.15 (0.57, 2.31)	0.69	0.01	66	R
	ID versus II		0.76 (0.41, 1.44)	0.40	0.001	75	R
	DD + ID versus II		0.86 (0.48, 1.55)	0.62	0.0009	76	R
	DD versus ID + II		1.27 (0.93, 1.72)	0.14	0.46	0	F
CVL	D versus I	6	1.10 (0.77, 1.56)	0.60	0.0006	77	R
	DD versus II		1.36 (0.71, 2.61)	0.35	0.01	66	R
	ID versus II		1.03 (0.56, 1.87)	0.93	0.0004	78	R
	DD + ID versus II		1.11(0.60, 2.03)	0.74	<0.0001	81	R
	DD versus ID + II		1.23 (0.92, 1.66)	0.16	0.33	13	F

### Correlation of ACE I/D polymorphism in severity of RHD

**Figure 3:**
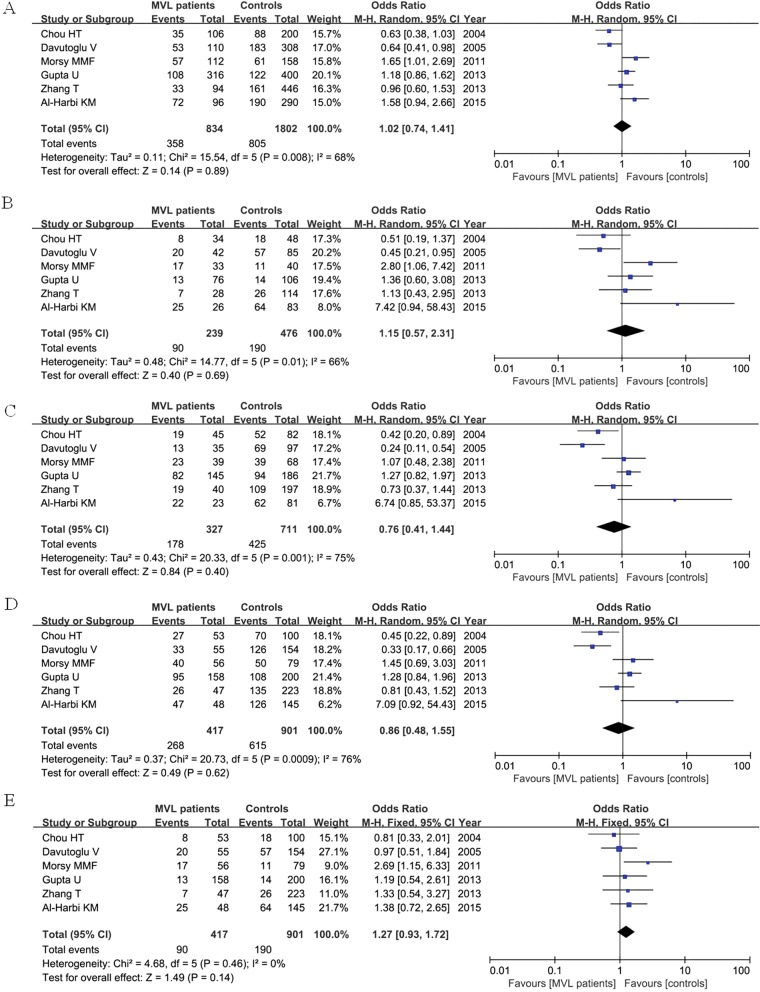
Forest plot of ACE I/D polymorphism in MVL RHD patients and controls under the five genetic models (**A:** D versus I; **B:** DD versus II; **C:** ID versus II; **D:** DD + ID versus II; **E:** DD versus ID + II)

**Figure 4:**
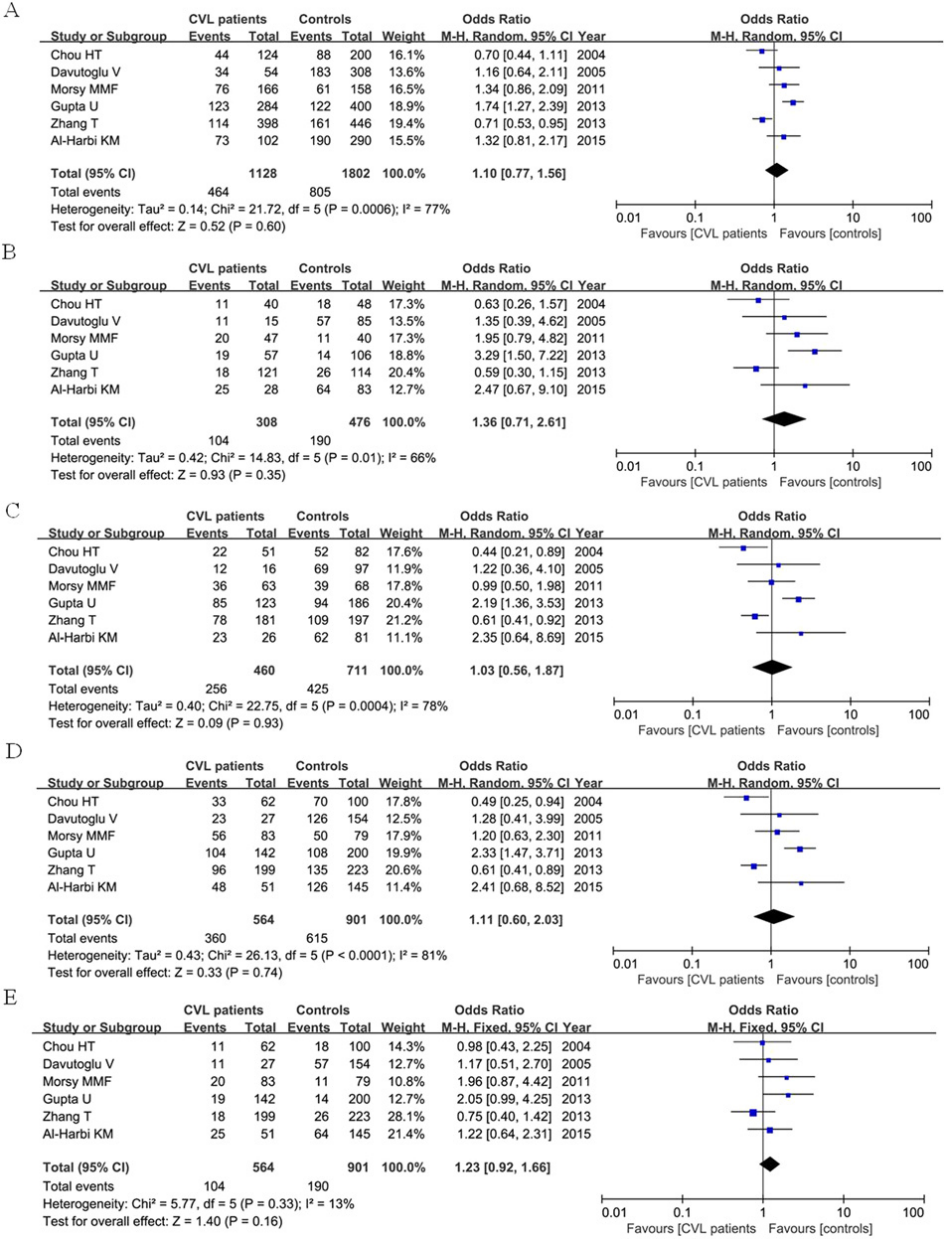
Meta-analysis of the association of ACE I/D polymorphism in CVL RHD patients and controls under the five genetic models (**A:** D versus I; **B:** DD versus II; **C:** ID versus II; **D:** DD + ID versus II; **E:** DD versus ID + II)

We divided the RHD patients into two groups according to valve involvement: mitral valve lesion (MVL) and combined valve lesion (CVL). Total six articles were included, containing 417 MVL patients, 564 CVL patients and 901 controls. For MVL group, our result found that ACE I/D polymorphism was not associated with increased risk of RHD patients with valve involvement when compared with control group under each genetic model as shown in Figure 3. For CVL group, no significant relationship was detected between ACE I/D polymorphism and CVL patients as well when compared with control group as shown in Figure 4.

### Correlation of ACE I/D polymorphism in RHD patients based on gender

**Figure 5:**
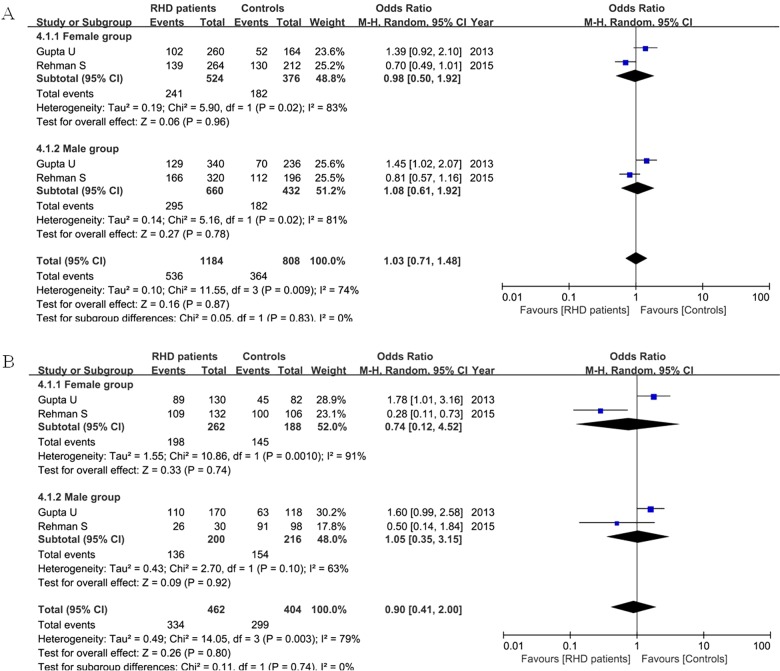
Meta-analysis of ACE I/D variant in female, male RHD patients and controls under the allelic model (**A:** D versus I) and dominant model (**B:** DD + ID versus II)

Two articles considered the gender issue. Our result did not find a significant association between female or male patients and the controls regarding the D allele (female: OR=0.98, 95% CI=0.50–1.90, *P*= 0.96; male: OR=1.08, 95% CI=0.61–1.92, *P*= 0.78) and DD + ID genotype (female: OR=0.74, 95% CI=0.12–4.52, *P*= 0.74; male: OR=1.05, 95% CI=0.35–3.15, *P*= 0.92) in the random-effect model as shown in Figure 5.

### Sensitivity analysis and publication bias

**Figure 6:**
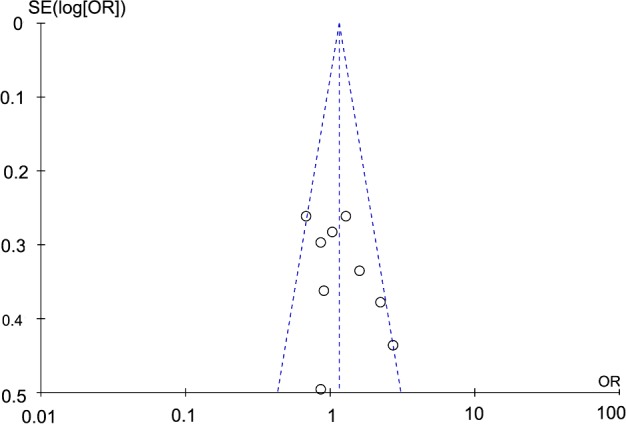
Funnel plot of ACE I/D polymorphism in RHD under the recessive model

A sensitivity analysis was performed to assess whether our results were influenced by the presence of each single study. Our result showed that the ORs were not significantly changed when we systematically deleted any individual study and recalculated the significance. The funnel plot was used to estimate the potential publication bias under each comparison model. The shape of the funnel plot did not reveal any obvious asymmetry as shown in Figure 6, indicating that there was no publication bias.

## DISCUSSION

In this meta-analysis, we totally screened out nine related articles. Overall, our result showed that ACE I/D variant was not associated with increased risk of RHD based on the existing research results. Subgroup analysis by valve involvement did not find a significant correlation between this genetic variant and MVL or CVL patients. Also, no significant difference was found between the female, male patients and the controls regarding ACE I/D polymorphism. Our result was inconformity with two previous meta-analyses: one was conducted by Gupta et al. [32] which only included four relevant articles and less participant (636 RHD cases and 533 controls), indicating that the ACE D allele was significantly associated with increased risk of RHD (*P*= 0.027); the other was conducted by Shang et al. [38] which contained a total of six articles (981 RHD patients and 901 controls), suggesting that the ACE I/D polymorphism was significantly associated with RHD, and DD genotype increases the risk of RHD (*P*= 0.04). The retrieved articles of these two meta-analyses were all included in our statistical analysis. This may due to the incidence of RHD and the distribution of ACE I/D polymorphism vary among different populations.

RHD is still prevalent in diverse regions of the world. Patients with RHD were predominantly young female, and had high prevalence of major cardiovascular complications, accounting for over a million premature deaths annually [39]. However, there is little contemporary information on presentation, complications and treatment of RHD. Several molecules that play a role in the immune response against the bacteria might be involved in the mechanisms leading to autoimmune reactions [40]. ACE is a zinc metallopeptidase, and makes important contributions to many different physiological processes [41,42]. Researches have discussed the functional significance of ACE tissue-specific expression and the presence in ACE of two independent catalytic sites with distinct substrates and biological effects [43]. ACE can act as the primary enzyme for the conversion of angiotensin-(1–12) to smaller angiotensin peptides in rodents [44]. Moreover, numerous potent domain-selective ACE inhibitors are available clinically, and generally effective in the treatment of hypertension, post-myocardial infarction and diabetic nephropathy [45]. The most notable ACE areRXP407 (N-domain) and RXPA380 (C-domain), which in principle may herald new therapeutic approaches for ACE inhibition [46].

It is known that the serum level of the ACE enzyme is stable within a given individual. But its concentration might be affected by genetic factors. ACE gene polymorphism might in cis influence the serum ACE level, and affect the expression of the ACE mRNA [47]. For example, the ACE IVS25+1G>A variant was shown to be associated with a major familial elevation of circulating ACE [48]; the deleted form of the ACE I/D variant (D allele) was associated with higher circulating and tissue ACE activity [49]. In addition, ACE I/D polymorphism was significantly related with serum ACE activity, and male subjects with DD genotype had higher serum ACE activity than female subjects with DD genotype in elderly Chinese [50]. ACE I/D polymorphism was one of the genetic factors for an inter-individual variability of brain substance *P* levels which might contribute to the susceptibility to affective disorders [51].

Some studies have hypothesized that the ACE I/D polymorphism was associated with RHD risk. However, the results remain inconclusive. In the retrieved articles of our meta-analysis, two studies from China, one study from Turkey and one study from Pakistan reported a significant relationship between the II genotype and RHD risk; one study from Kazakhstan did not find an association between ACE I/D variant and RHD; other four studies have demonstrated a correlation between DD genotype and RHD susceptibility. Our statistical analysis did not find a significant association between ACE I/D polymorphism and RHD risk under any genetic models. There are two reasons to explain the result. The first one is that the incidence of RHD and the distribution of ACE I/D polymorphism vary among different participants. The second one is that there are less included studies. ACE I/D polymorphism might be associated with other disease risk. Evidences have shown that D allele of ACE I/D polymorphism was associated with increased risk of end-stage renal disease susceptibility [52], coronary artery disease in T2DM patients [53] and early onset primary knee osteoarthritis in Asian Indian populations [54]. This genetic variant is also a low-penetrance susceptibility marker of ischaemic stroke [55].

Several limitations were presented in our study. Firstly, there was moderate between-study heterogeneity among all the genetic models except the recessive model, and the genotype distribution showed deviation from HWE in one study. Secondly, most of the retrieved articles for the ACE variant in RHD patients were conducted in Asian populations, which limited the statistical power to detect the association between this genetic polymorphism and RHD risk among other ethnicities. Thirdly, some important confounding effectors, such as age, sex, family history of rheumatic fever were unable to be extracted from each included study. Lastly, gene–gene and gene–environment interaction should be considered in the future researches.

In conclusions, the present meta-analysis indicates that existing research results are still not enough to prove the link between ACE I/D polymorphism and RHD. Future well-designed studies with more ethnicities are still required to further evaluate the effect of ACE variant on RHD risk.

## Abbreviations

ACE: angiotensin-converting enzyme
ACE I/D: angiotensin I-converting enzyme gene insertion/deletion polymorphism
Ang: angiotensin
CI: confidence interval
CVL: combined valve lesion
HWE: Hardy–Weinberg equilibrium
MVL: mitral valve lesion
OR: odds ratio
RAS: renin–angiotensin system
RHD: rheumatic heart disease
